# Supplementary material for: The Effects of Patient-Reported Outcome Screening on the Survival of People with Cancer: A Systematic Review and Meta-Analysis
Source: Cancers (Basel). 2022 Nov 7;14(21):5470. doi: 10.3390/cancers14215470 (PMC9657884; doi:10.3390/cancers14215470)
Supplement: Supplementary file 1 [file cancers-14-05470-s001.zip › cancers-1998221-supplementary.pdf]

Supplementary

The Effects of Patient-Reported Outcome Screening on the Survival of People with Cancer: A Systematic Review and Meta-Analysis

Table S1. Studies excluded after full text review and corresponding reasons.

FIRST AUTHOR	YEAR	REASON FOR EXCLUSION
de Rooij	2018	Study aims not relevant to the review
Geerse	2017	Study intervention not restricted to PRO screening
Gilbert	2015	Outcome of interest not measured
Henschel	2020	Controls also receive an intervention
Oerlemans	2021	Outcome of interest not measured
Skovlund	2021	Outcome of interest not measured

Table S2. Risk of bias summaries for randomized trials and non-randomized trials of interventions.

(A). Risk of bias summary for RCTs: review authors' judgements about each risk of bias item for each included study.

Author (year)	Risk of bias arising from the randomization process	Risk of bias due to deviations from the intended interventions (effect of assignment to intervention)	Risk of bias due to missing outcome data	Risk of bias in measurement of the outcome	Risk of bias in the selection of the reported result	Overall risk of bias
Bash 2016	LOW Randomisation was conducted by the institutional Biostatistics Service via a computer system	SOME CONCERNS Participants were aware that they were in a study, but no deviations or possible influences are reported; it is not indicated whether intention-to-treat (ITT) analyses were carried out.	LOW We judge the risk of bias as low, even though the number of missing data and its impact on the survival outcome in the two groups are not clearly indicated.	LOW Survival was tabulated for both groups based on medical records and social security Death Index data.	SOME CONCERNS Survival was a secondary end point of the RCT, and corresponding results (OR and confidence interval) of the logistic analysis foreseen in the statistical analysis plan are not clearly reported. A post-hoc analysis on overall survival was published in a subsequent paper	**SOME CONCERNS** The study is judged to raise some concerns in two domains, mostly due to the type of study with PROs and to the use of post hoc analysis for the outcome of interest

					[Basch 2017] after a median follow-up of 7 years. In this secondary analysis the results of the multivariate analysis are reported only in part (overall HR, Confidence Interval and significance), omitting information regarding the role of the variables included in the model.	
Denis 2017	SOME CONCERNS<br>We judge the risk of bias as moderate, as randomisation was performed using statistical software, but allocation concealment is not indicated	LOW<br>Participants were aware that they were in a study, but no deviations or possible influences before interim analysis leading to study termination are reported.<br>Intention-to-treat (ITT) analyses were undertaken.	LOW<br>The number of missing data and its impact on the survival outcome are not indicated. Figure 3 seems to suggest that there were no missing data for the survival end point.	LOW<br>Survival data were gathered from patient follow-up by blinded investigators.	SOME CONCERNS<br>The overall number of patients (60% of the prespecified sample size) is small because the interim analysis led to early trial stoppage<br>due to the large survival benefit observed in the experimental arm.<br>Results of clinical follow-up two years after randomization were published subsequently [Denis 2019], but are not reported in detail.	**SOME CONCERNS**<br>The study is judged to raise some concerns in two domains, mostly due to the type of study with PROs and to early stoppage following interim analysis

(B). Risk of bias summary for Non-randomized Studies of Intervention (NRS): review authors' judgements about each risk of bias item for each included study.

STUDY TYPE: NRS								
**Author (year)**	**Bias due to confounding**	**Bias in selection of participants into study**	**Bias in classification of interventions**	**Bias due to deviations from intended intervention**	**Bias due to missing data**	**Bias in measurement of outcomes**	**Bias in selection of the reported result**	**Overall risk of bias**
Barbera 2020	MODERATE Although the two groups were matched, comparison of baseline characteristics highlights differences which appear to have influenced intervention administration. The data may be missing important clinical prognostic information which may be different between the two groups.	LOW We judge risk of bias in this domain to be low, although subjects were retrospectively reviewed and patients with invalid or missing information on demographics were excluded.	LOW Interventions were well defined	LOW No deviations appear to be present.	LOW No lost to follow up are reported.	LOW No bias in outcome data detection appears to be present.	LOW All of the indicators mentioned in the methodology are reported.	**LOW** We judge potential risk of bias as low, also in the light of the large sample size
Demedts 2021	MODERATE Baseline differences between the 2 groups are not reported. Since group assignment was performed following patient choice, there may be one or more prognostic factors unequally distributed.	MODERATE Survival was assessed in a subgroup of stage IV Non Small Cell Lung Cancer patients	LOW The intervention was thoroughly described in a previous paper. Standard of care is not described.	LOW No deviations from the intervention appear to be present which may have influenced mortality.	LOW No lost to follow-up are reported.	LOW No bias in outcome data detection appears to be present.	MODERATE The paper does not report mortality data for all included patients,nor results of the multivariate Cox regression analysis.	**MODERATE** The study exhibits problems in 3 domains
Patel 2019	SERIOUS The study is single-centered, and the intervention arm comprises more stage IV patients than the control arm.	MODERATE Patients in the intervention group were enrolled prospectively, whereas a historical cohort was used as the control group	LOW Classification of intervention is clear, since groups refer to two distinct periods of time	LOW No deviations from the intervention appear to be present which may have influenced mortality.	LOW No lost to follow up are reported.	LOW Mortality data were collected using two official validated registries.	MODERATE Data on the two survival curves and on risk of death using Cox proportional hazards regression models are	**SERIOUS** Baseline differences between the two arms may have influenced overall mortality

							not adequately reported.	
Patel 2020	LOW The study is multicentered and no baseline differences are observed between the characteristics of patients in the two arms	MODERATE Patients in the intervention group were enrolled prospectively, whereas a historical cohort was used as the control group	LOW Classification of intervention is clear, since groups refer to two distinct periods of time	LOW No deviations from the intervention appear to be present which may have influenced mortality	LOW No lost to follow up are reported.	LOW Mortality data were collected using two official validated registries.	MODERATE Data on the two survival curves and on risk of death using Cox proportional hazards regression are not adequately reported.	**MODERATE** The study exhibits problems in two domains

A) Risk of bias for non-randomised studies (NRS) based on ROBINS-I tool [Sterne J A, HernÃ¡n M A, Reeves B C, SavoviÄ‡ J, Berkman N D, Viswanathan M et al. ROBINS-I: a tool for assessing risk of bias in non-randomised studies of interventions BMJ 2016; 355 :i4919 doi:10.1136/bmj.i4919].
B) Risk of bias for randomized trials (RCT) based on the Cochrane Collaboration tool (Rob 2 - version 2.0) [Sterne JAC, Savović J, Page MJ, Elbers RG, Blencowe NS, Boutron I, et al. RoB 2: a revised tool for assessing risk of bias in randomised trials. BMJ. 2019;366. 10.1136/bmj.l4898].

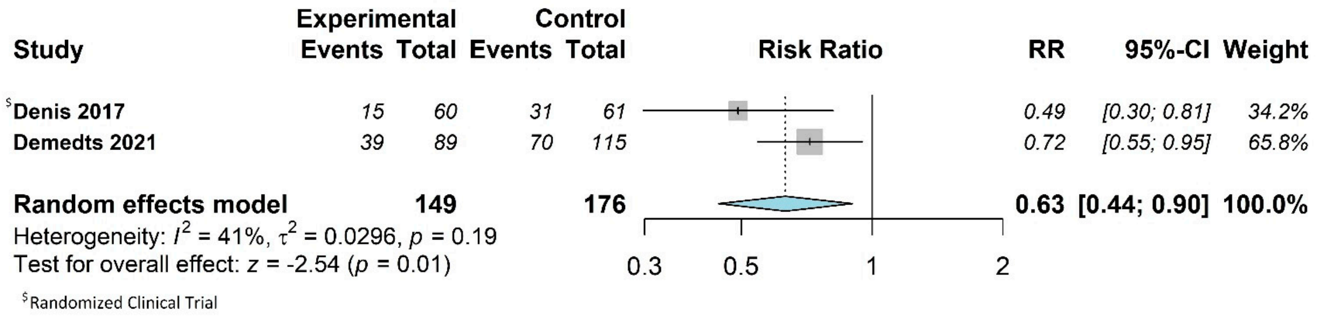

Figure S1. Subgroup analysis for lung cancer shown as a forest plot of the survival pooled effect.

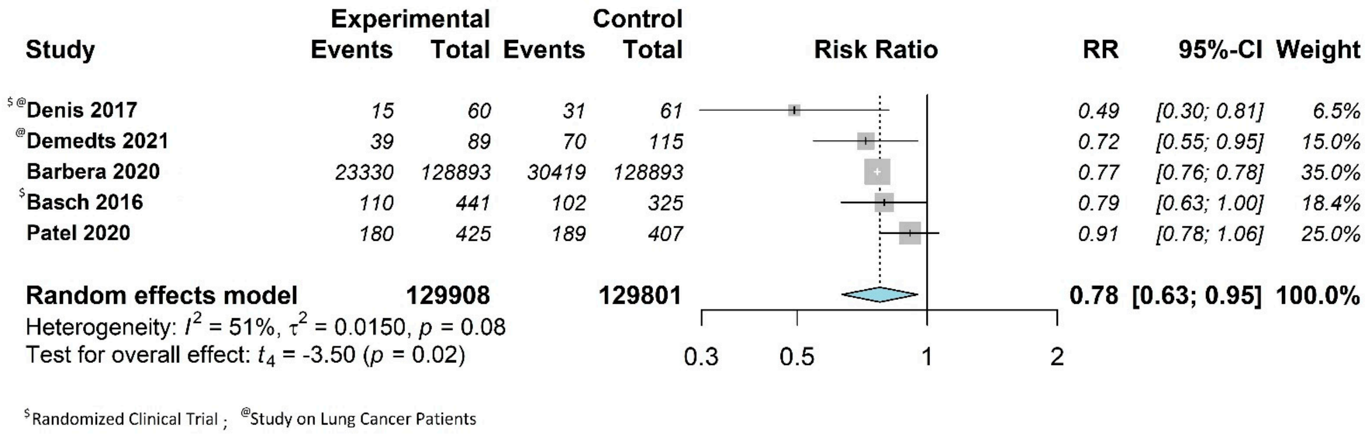

Figure S2. Sensitivity analysis shown as a forest plot of the survival pooled effect excluding one study with the overall risk of bias rated as serious.
